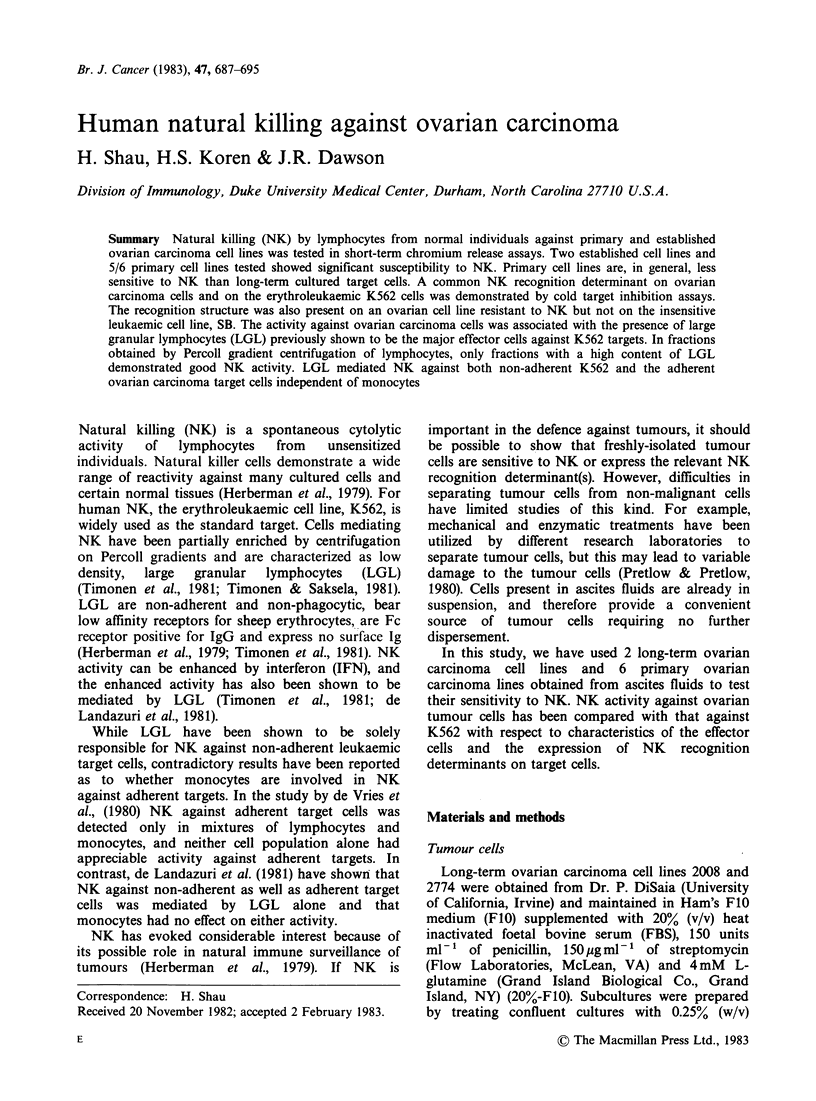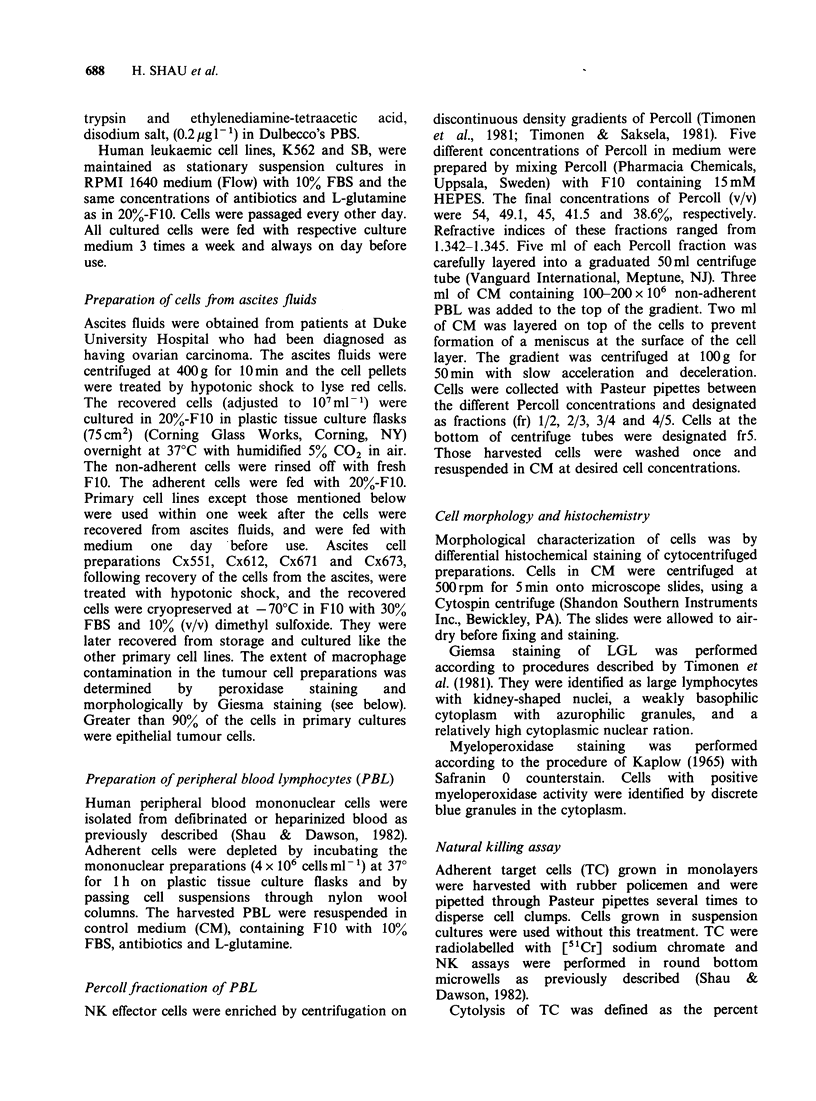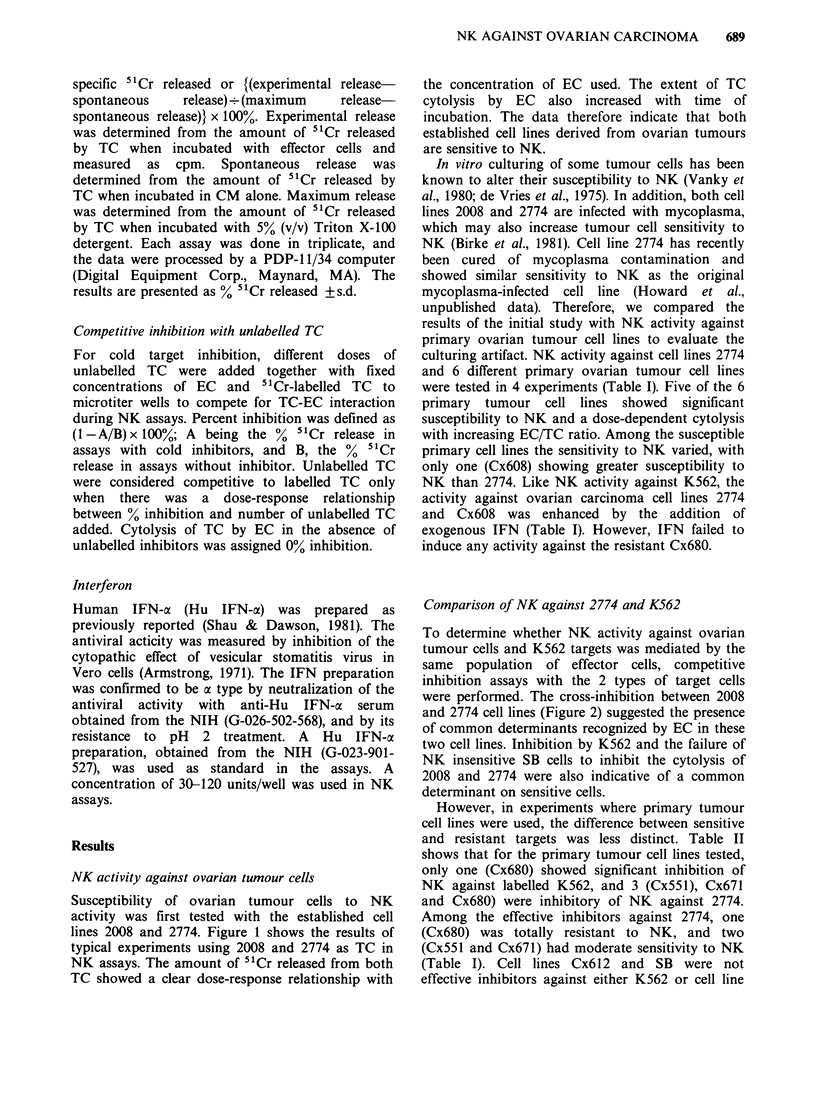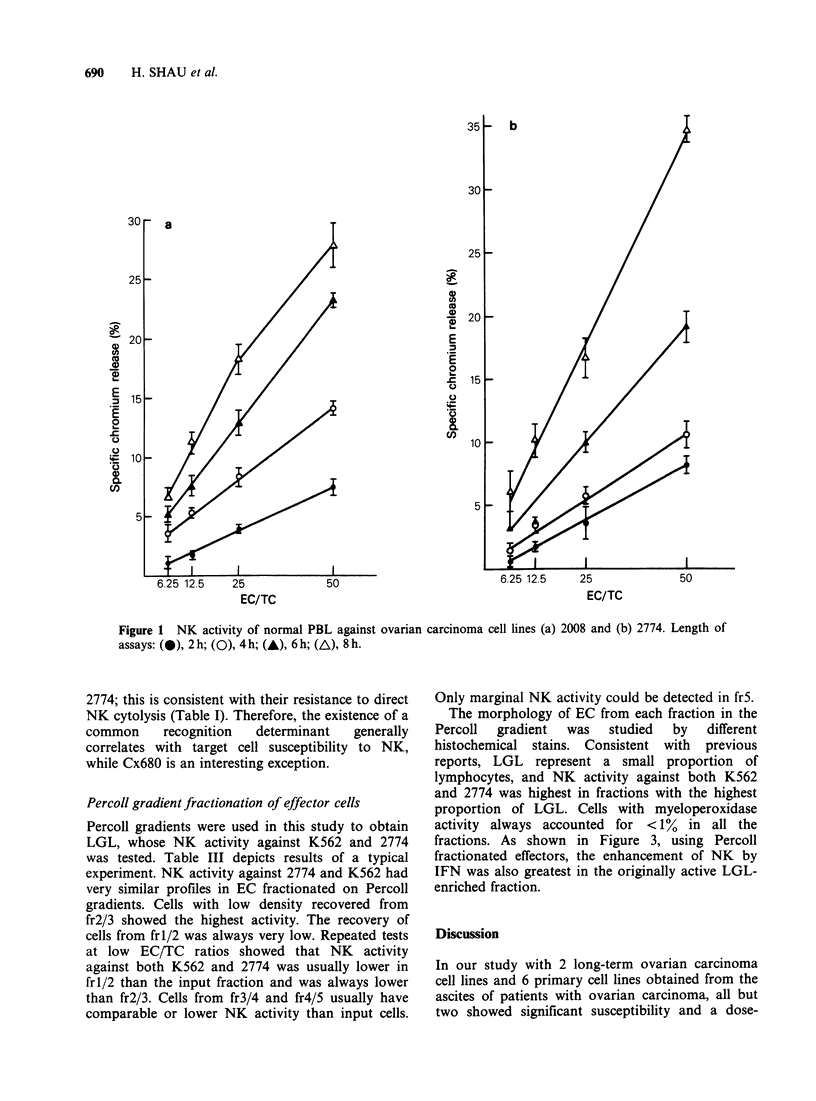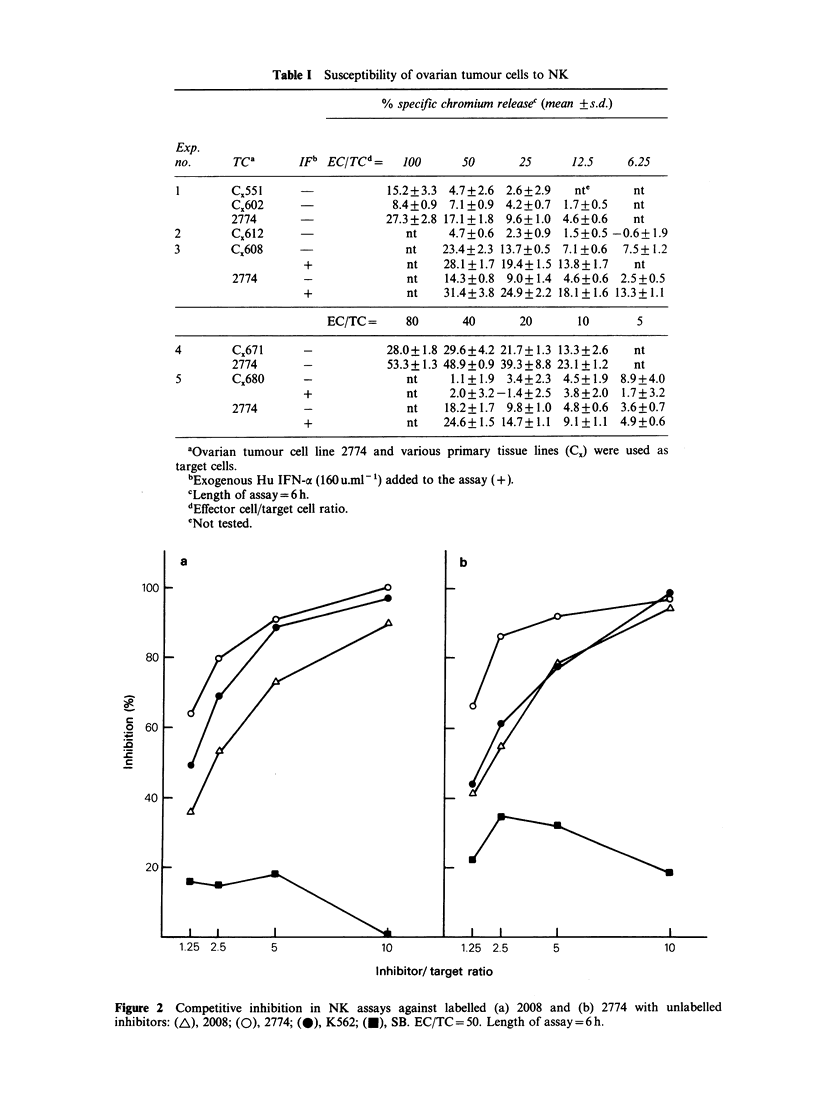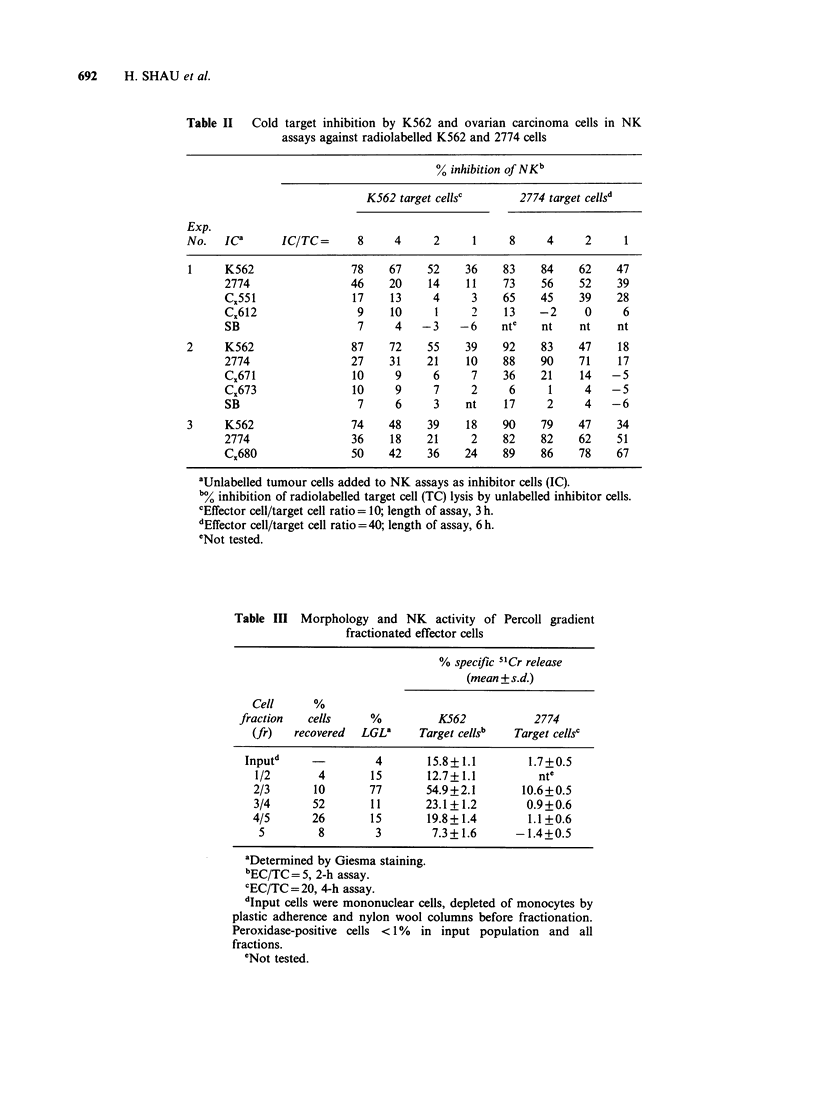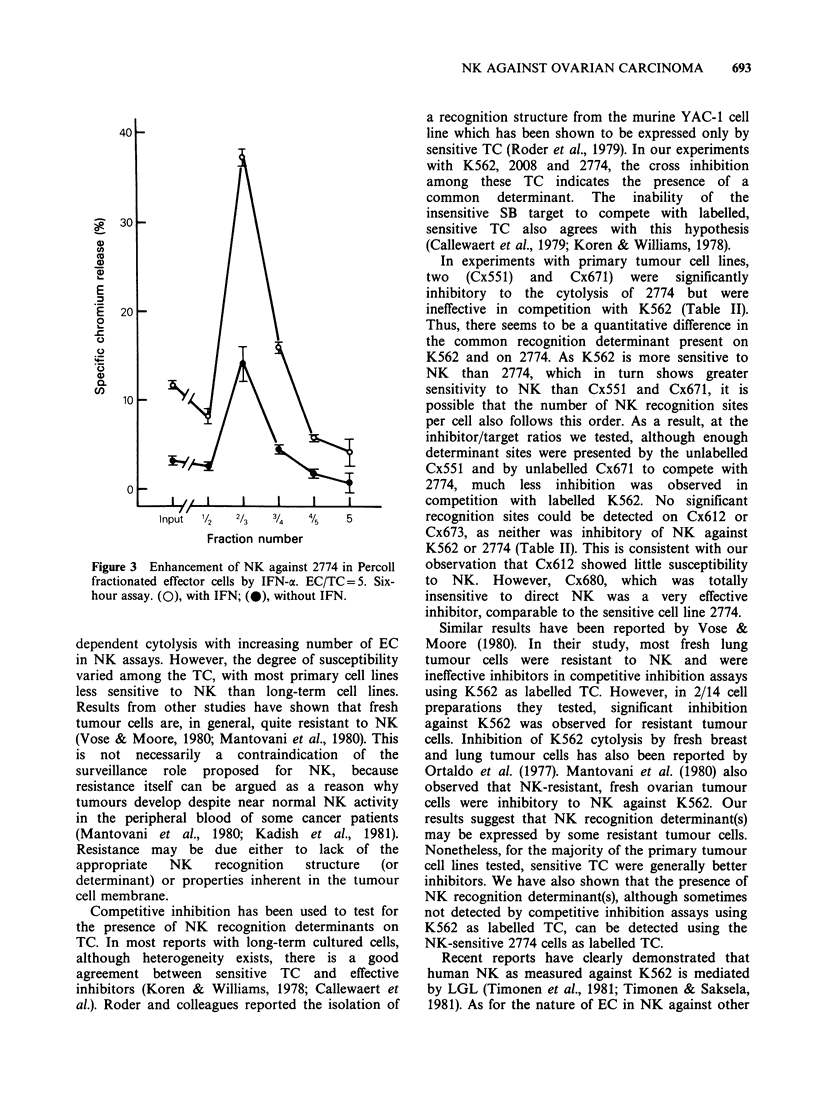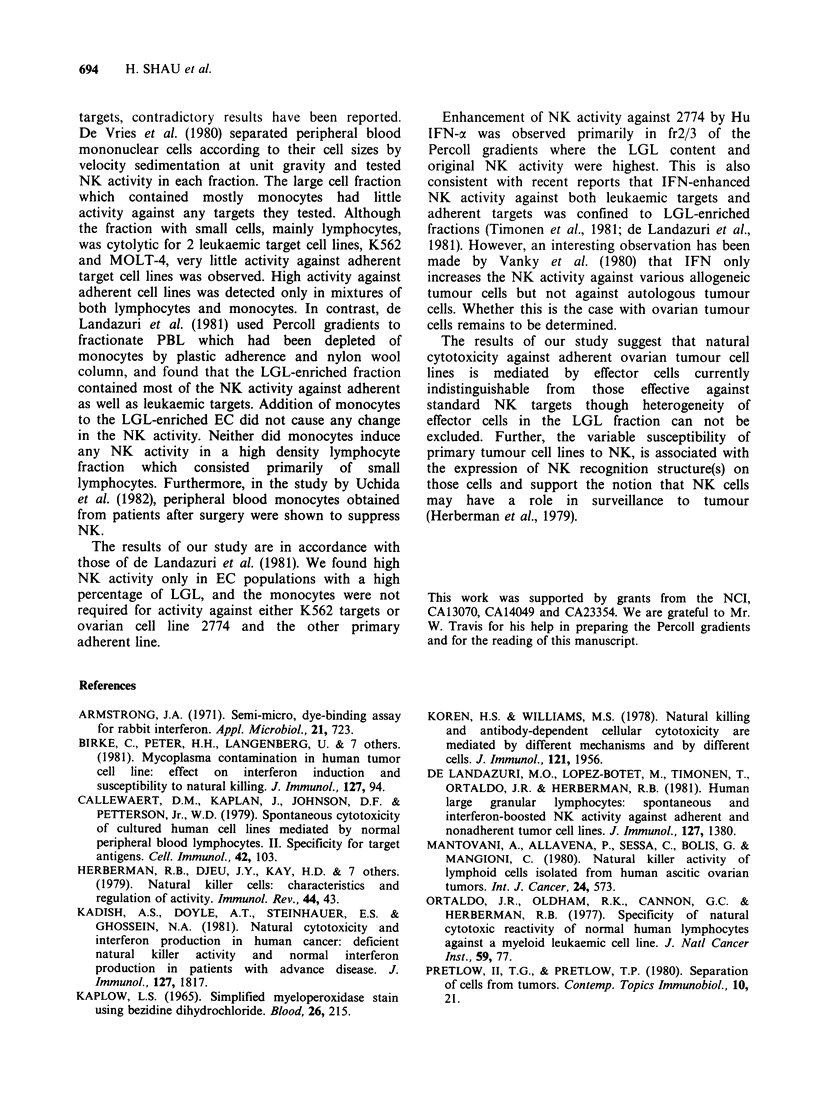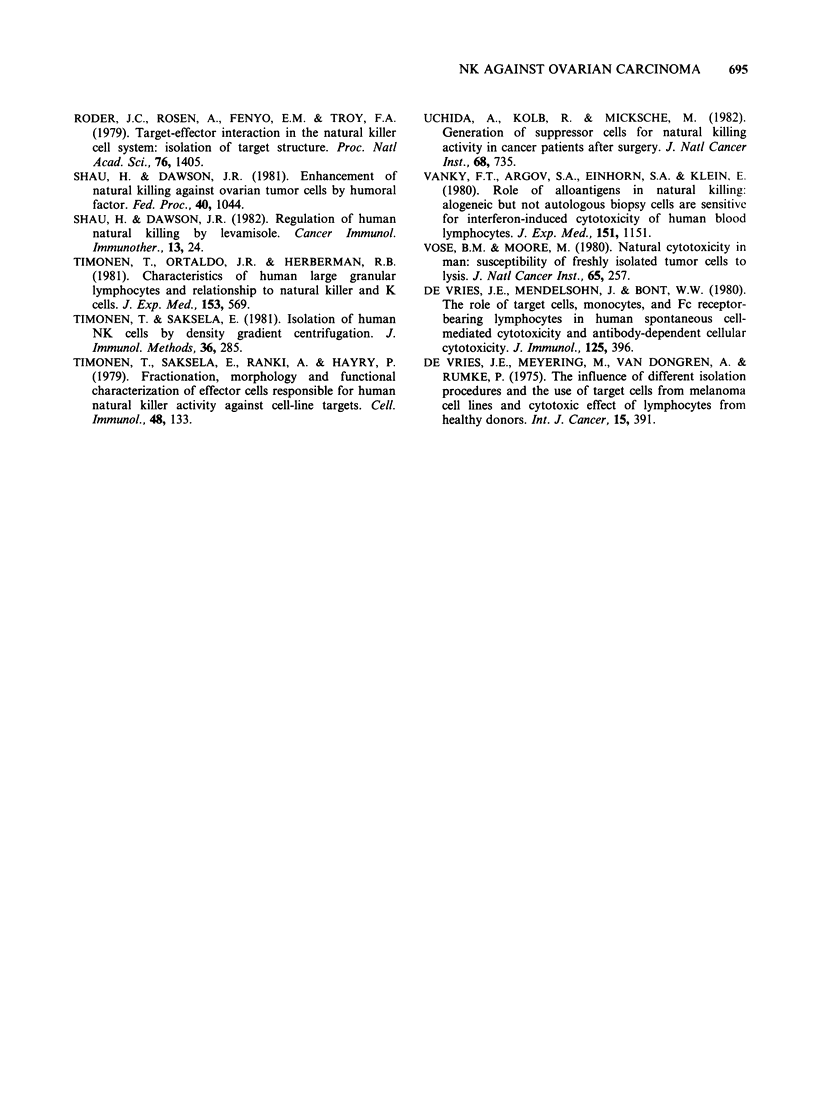# Human natural killing against ovarian carcinoma.

**DOI:** 10.1038/bjc.1983.107

**Published:** 1983-05

**Authors:** H. Shau, H. S. Koren, J. R. Dawson

## Abstract

Natural killing (NK) by lymphocytes from normal individuals against primary and established ovarian carcinoma cell lines was tested in short-term chromium release assays. Two established cell lines and 5/6 primary cell lines tested showed significant susceptibility to NK. Primary cell lines are, in general, less sensitive to NK than long-term cultured target cells. A common NK recognition determinant on ovarian carcinoma cells and on the erythroleukaemic K562 cells was demonstrated by cold target inhibition assays. The recognition structure was also present on an ovarian cell line resistant to NK but not on the insensitive leukaemic cell line, SB. The activity against ovarian carcinoma cells was associated with the presence of large granular lymphocytes (LGL) previously shown to be the major effector cells against K562 targets. In fractions obtained by Percoll gradient centrifugation of lymphocytes, only fractions with a high content of LGL demonstrated good NK activity. LGL mediated NK against both non-adherent K562 and the adherent ovarian carcinoma target cells independent of monocytes.


Br. J. Cancer (1983), 47, 687–695

### Human natural killing against ovarian carcinoma

H. Shau, H.S. Koren & J.R. Dawson

*Division of Immunology, Duke University Medical Center, Durham, North Carolina 27710 U.S.A.*

**Summary** Natural killing (NK) by lymphocytes from normal individuals against primary and established ovarian carcinoma cell lines was tested in short-term chromium release assays. Two established cell lines and 5/6 primary cell lines tested showed significant susceptibility to NK. Primary cell lines are, in general, less sensitive to NK than long-term cultured target cells. A common NK recognition determinant on ovarian carcinoma cells and on the erythroleukaemic K562 cells was demonstrated by cold target inhibition assays. The recognition structure was also present on an ovarian cell line resistant to NK but not on the insensitive leukaemic cell line, SB. The activity against ovarian carcinoma cells was associated with the presence of large granular lymphocytes (LGL) previously shown to be the major effector cells against K562 targets. In fractions obtained by Percoll gradient centrifugation of lymphocytes, only fractions with a high content of LGL demonstrated good NK activity. LGL mediated NK against both non-adherent K562 and the adherent ovarian carcinoma target cells independent of monocytes

Natural killing (NK) is a spontaneous cytolytic activity of lymphocytes from unsensitized individuals. Natural killer cells demonstrate a wide range of reactivity against many cultured cells and certain normal tissues (Herberman *et al.*, 1979). For human NK, the erythroleukaemic cell line, K562, is widely used as the standard target. Cells mediating NK have been partially enriched by centrifugation on Percoll gradients and are characterized as low density, large granular lymphocytes (LGL) (Timonen *et al.*, 1981; Timonen & Saksela, 1981). LGL are non-adherent and non-phagocytic, bear low affinity receptors for sheep erythrocytes, are Fc receptor positive for IgG and express no surface Ig (Herberman *et al.*, 1979; Timonen *et al.*, 1981). NK activity can be enhanced by interferon (IFN), and the enhanced activity has also been shown to be mediated by LGL (Timonen *et al.*, 1981; de Landazuri *et al.*, 1981).

While LGL have been shown to be solely responsible for NK against non-adherent leukaemic target cells, contradictory results have been reported as to whether monocytes are involved in NK against adherent targets. In the study by de Vries *et al.*, (1980) NK against adherent target cells was detected only in mixtures of lymphocytes and monocytes, and neither cell population alone had appreciable activity against adherent targets. In contrast, de Landazuri *et al.* (1981) have shown that NK against non-adherent as well as adherent target cells was mediated by LGL alone and that monocytes had no effect on either activity.

NK has evoked considerable interest because of its possible role in natural immune surveillance of tumours (Herberman *et al.*, 1979). If NK is important in the defence against tumours, it should be possible to show that freshly-isolated tumour cells are sensitive to NK or express the relevant NK recognition determinant(s). However, difficulties in separating tumour cells from non-malignant cells have limited studies of this kind. For example, mechanical and enzymatic treatments have been utilized by different research laboratories to separate tumour cells, but this may lead to variable damage to the tumour cells (Pretlow & Pretlow, 1980). Cells present in ascites fluids are already in suspension, and therefore provide a convenient source of tumour cells requiring no further dispersement.

In this study, we have used 2 long-term ovarian carcinoma cell lines and 6 primary ovarian carcinoma lines obtained from ascites fluids to test their sensitivity to NK. NK activity against ovarian tumour cells has been compared with that against K562 with respect to characteristics of the effector cells and the expression of NK recognition determinants on target cells.

### Materials and methods

### *Tumour cells*

Long-term ovarian carcinoma cell lines 2008 and 2774 were obtained from Dr. P. DiSaia (University of California, Irvine) and maintained in Ham's F10 medium (F10) supplemented with 20% (v/v) heat inactivated foetal bovine serum (FBS), 150 units $ml^{-1}$ of penicillin, 150 $\mu g\,ml^{-1}$ of streptomycin (Flow Laboratories, McLean, VA) and 4 mM L-glutamine (Grand Island Biological Co., Grand Island, NY) (20%-F10). Subcultures were prepared by treating confluent cultures with 0.25% (w/v)

Correspondence: H. Shau
Received 20 November 1982; accepted 2 February 1983.

© The Macmillan Press Ltd., 1983

trypsin and ethylenediamine-tetraacetic acid, disodium salt, ($0.2\,\mu g\,l^{-1}$) in Dulbecco's PBS.

Human leukaemic cell lines, K562 and SB, were maintained as stationary suspension cultures in RPMI 1640 medium (Flow) with 10% FBS and the same concentrations of antibiotics and L-glutamine as in 20%-F10. Cells were passaged every other day. All cultured cells were fed with respective culture medium 3 times a week and always on day before use.

*Preparation of cells from ascites fluids*

Ascites fluids were obtained from patients at Duke University Hospital who had been diagnosed as having ovarian carcinoma. The ascites fluids were centrifuged at 400 g for 10 min and the cell pellets were treated by hypotonic shock to lyse red cells. The recovered cells (adjusted to $10^7\,ml^{-1}$) were cultured in 20%-F10 in plastic tissue culture flasks ($75\,cm^2$) (Corning Glass Works, Corning, NY) overnight at 37°C with humidified 5% $CO_2$ in air. The non-adherent cells were rinsed off with fresh F10. The adherent cells were fed with 20%-F10. Primary cell lines except those mentioned below were used within one week after the cells were recovered from ascites fluids, and were fed with medium one day before use. Ascites cell preparations Cx551, Cx612, Cx671 and Cx673, following recovery of the cells from the ascites, were treated with hypotonic shock, and the recovered cells were cryopreserved at −70°C in F10 with 30% FBS and 10% (v/v) dimethyl sulfoxide. They were later recovered from storage and cultured like the other primary cell lines. The extent of macrophage contamination in the tumour cell preparations was determined by peroxidase staining and morphologically by Giesma staining (see below). Greater than 90% of the cells in primary cultures were epithelial tumour cells.

*Preparation of peripheral blood lymphocytes (PBL)*

Human peripheral blood mononuclear cells were isolated from defibrinated or heparinized blood as previously described (Shau & Dawson, 1982). Adherent cells were depleted by incubating the mononuclear preparations ($4\times10^6$ cells $ml^{-1}$) at 37° for 1 h on plastic tissue culture flasks and by passing cell suspensions through nylon wool columns. The harvested PBL were resuspended in control medium (CM), containing F10 with 10% FBS, antibiotics and L-glutamine.

*Percoll fractionation of PBL*

NK effector cells were enriched by centrifugation on discontinuous density gradients of Percoll (Timonen et al., 1981; Timonen & Saksela, 1981). Five different concentrations of Percoll in medium were prepared by mixing Percoll (Pharmacia Chemicals, Uppsala, Sweden) with F10 containing 15 mM HEPES. The final concentrations of Percoll (v/v) were 54, 49.1, 45, 41.5 and 38.6%, respectively. Refractive indices of these fractions ranged from 1.342–1.345. Five ml of each Percoll fraction was carefully layered into a graduated 50 ml centrifuge tube (Vanguard International, Meptune, NJ). Three ml of CM containing 100–200 $\times 10^6$ non-adherent PBL was added to the top of the gradient. Two ml of CM was layered on top of the cells to prevent formation of a meniscus at the surface of the cell layer. The gradient was centrifuged at 100 g for 50 min with slow acceleration and deceleration. Cells were collected with Pasteur pipettes between the different Percoll concentrations and designated as fractions (fr) 1/2, 2/3, 3/4 and 4/5. Cells at the bottom of centrifuge tubes were designated fr5. Those harvested cells were washed once and resuspended in CM at desired cell concentrations.

*Cell morphology and histochemistry*

Morphological characterization of cells was by differential histochemical staining of cytocentrifuged preparations. Cells in CM were centrifuged at 500 rpm for 5 min onto microscope slides, using a Cytospin centrifuge (Shandon Southern Instruments Inc., Bewickley, PA). The slides were allowed to air-dry before fixing and staining.

Giemsa staining of LGL was performed according to procedures described by Timonen et al. (1981). They were identified as large lymphocytes with kidney-shaped nuclei, a weakly basophilic cytoplasm with azurophilic granules, and a relatively high cytoplasmic nuclear ration.

Myeloperoxidase staining was performed according to the procedure of Kaplow (1965) with Safranin 0 counterstain. Cells with positive myeloperoxidase activity were identified by discrete blue granules in the cytoplasm.

*Natural killing assay*

Adherent target cells (TC) grown in monolayers were harvested with rubber policemen and were pipetted through Pasteur pipettes several times to disperse cell clumps. Cells grown in suspension cultures were used without this treatment. TC were radiolabelled with [$^{51}$Cr] sodium chromate and NK assays were performed in round bottom microwells as previously described (Shau & Dawson, 1982).

Cytolysis of TC was defined as the percent specific $^{51}Cr$ released or {(experimental release—spontaneous release)÷(maximum release—spontaneous release)} × 100%. Experimental release was determined from the amount of $^{51}Cr$ released by TC when incubated with effector cells and measured as cpm. Spontaneous release was determined from the amount of $^{51}Cr$ released by TC when incubated in CM alone. Maximum release was determined from the amount of $^{51}Cr$ released by TC when incubated with 5% (v/v) Triton X-100 detergent. Each assay was done in triplicate, and the data were processed by a PDP-11/34 computer (Digital Equipment Corp., Maynard, MA). The results are presented as % $^{51}Cr$ released ±s.d.

*Competitive inhibition with unlabelled TC*

For cold target inhibition, different doses of unlabelled TC were added together with fixed concentrations of EC and $^{51}Cr$-labelled TC to microtiter wells to compete for TC-EC interaction during NK assays. Percent inhibition was defined as $(1-A/B) \times 100\%$; A being the % $^{51}Cr$ release in assays with cold inhibitors, and B, the % $^{51}Cr$ release in assays without inhibitor. Unlabelled TC were considered competitive to labelled TC only when there was a dose-response relationship between % inhibition and number of unlabelled TC added. Cytolysis of TC by EC in the absence of unlabelled inhibitors was assigned 0% inhibition.

*Interferon*

Human IFN-α (Hu IFN-α) was prepared as previously reported (Shau & Dawson, 1981). The antiviral acticity was measured by inhibition of the cytopathic effect of vesicular stomatitis virus in Vero cells (Armstrong, 1971). The IFN preparation was confirmed to be α type by neutralization of the antiviral activity with anti-Hu IFN-α serum obtained from the NIH (G-026-502-568), and by its resistance to pH 2 treatment. A Hu IFN-α preparation, obtained from the NIH (G-023-901-527), was used as standard in the assays. A concentration of 30–120 units/well was used in NK assays.

### Results

*NK activity against ovarian tumour cells*

Susceptibility of ovarian tumour cells to NK activity was first tested with the established cell lines 2008 and 2774. Figure 1 shows the results of typical experiments using 2008 and 2774 as TC in NK assays. The amount of $^{51}Cr$ released from both TC showed a clear dose-response relationship with the concentration of EC used. The extent of TC cytolysis by EC also increased with time of incubation. The data therefore indicate that both established cell lines derived from ovarian tumours are sensitive to NK.

*In vitro* culturing of some tumour cells has been known to alter their susceptibility to NK (Vanky *et al.*, 1980; de Vries *et al.*, 1975). In addition, both cell lines 2008 and 2774 are infected with mycoplasma, which may also increase tumour cell sensitivity to NK (Birke *et al.*, 1981). Cell line 2774 has recently been cured of mycoplasma contamination and showed similar sensitivity to NK as the original mycoplasma-infected cell line (Howard *et al.*, unpublished data). Therefore, we compared the results of the initial study with NK activity against primary ovarian tumour cell lines to evaluate the culturing artifact. NK activity against cell lines 2774 and 6 different primary ovarian tumour cell lines were tested in 4 experiments (Table I). Five of the 6 primary tumour cell lines showed significant susceptibility to NK and a dose-dependent cytolysis with increasing EC/TC ratio. Among the susceptible primary cell lines the sensitivity to NK varied, with only one (Cx608) showing greater susceptibility to NK than 2774. Like NK activity against K562, the activity against ovarian carcinoma cell lines 2774 and Cx608 was enhanced by the addition of exogenous IFN (Table I). However, IFN failed to induce any activity against the resistant Cx680.

*Comparison of NK against 2774 and K562*

To determine whether NK activity against ovarian tumour cells and K562 targets was mediated by the same population of effector cells, competitive inhibition assays with the 2 types of target cells were performed. The cross-inhibition between 2008 and 2774 cell lines (Figure 2) suggested the presence of common determinants recognized by EC in these two cell lines. Inhibition by K562 and the failure of NK insensitive SB cells to inhibit the cytolysis of 2008 and 2774 were also indicative of a common determinant on sensitive cells.

However, in experiments where primary tumour cell lines were used, the difference between sensitive and resistant targets was less distinct. Table II shows that for the primary tumour cell lines tested, only one (Cx680) showed significant inhibition of NK against labelled K562, and 3 (Cx551), Cx671 and Cx680) were inhibitory of NK against 2774. Among the effective inhibitors against 2774, one (Cx680) was totally resistant to NK, and two (Cx551 and Cx671) had moderate sensitivity to NK (Table I). Cell lines Cx612 and SB were not effective inhibitors against either K562 or cell line

**Figure 1** NK activity of normal PBL against ovarian carcinoma cell lines (a) 2008 and (b) 2774. Length of assays: (●), 2 h; (○), 4 h; (▲), 6 h; (△), 8 h.

2774; this is consistent with their resistance to direct NK cytolysis (Table I). Therefore, the existence of a common recognition determinant generally correlates with target cell susceptibility to NK, while Cx680 is an interesting exception.

*Percoll gradient fractionation of effector cells*

Percoll gradients were used in this study to obtain LGL, whose NK activity against K562 and 2774 was tested. Table III depicts results of a typical experiment. NK activity against 2774 and K562 had very similar profiles in EC fractionated on Percoll gradients. Cells with low density recovered from fr2/3 showed the highest activity. The recovery of cells from fr1/2 was always very low. Repeated tests at low EC/TC ratios showed that NK activity against both K562 and 2774 was usually lower in fr1/2 than the input fraction and was always lower than fr2/3. Cells from fr3/4 and fr4/5 usually have comparable or lower NK activity than input cells. Only marginal NK activity could be detected in fr5.

The morphology of EC from each fraction in the Percoll gradient was studied by different histochemical stains. Consistent with previous reports, LGL represent a small proportion of lymphocytes, and NK activity against both K562 and 2774 was highest in fractions with the highest proportion of LGL. Cells with myeloperoxidase activity always accounted for <1% in all the fractions. As shown in Figure 3, using Percoll fractionated effectors, the enhancement of NK by IFN was also greatest in the originally active LGL-enriched fraction.

### Discussion

In our study with 2 long-term ovarian carcinoma cell lines and 6 primary cell lines obtained from the ascites of patients with ovarian carcinoma, all but two showed significant susceptibility and a dose-

**Table I** Susceptibility of ovarian tumour cells to NK

| *Exp. no.* | *TC*[a] | *IF*[b] | *EC/TC*[d] = | % specific chromium release[c] (mean ±s.d.) 100 | 50 | 25 | 12.5 | 6.25 |
|---|---|---|---|---|---|---|---|---|
| 1 | $C_x$551 | — | | 15.2±3.3 | 4.7±2.6 | 2.6±2.9 | nt[e] | nt |
| | $C_x$602 | — | | 8.4±0.9 | 7.1±0.9 | 4.2±0.7 | 1.7±0.5 | nt |
| | 2774 | — | | 27.3±2.8 | 17.1±1.8 | 9.6±1.0 | 4.6±0.6 | nt |
| 2 | $C_x$612 | — | | nt | 4.7±0.6 | 2.3±0.9 | 1.5±0.5 | −0.6±1.9 |
| 3 | $C_x$608 | — | | nt | 23.4±2.3 | 13.7±0.5 | 7.1±0.6 | 7.5±1.2 |
| | | + | | nt | 28.1±1.7 | 19.4±1.5 | 13.8±1.7 | nt |
| | 2774 | − | | nt | 14.3±0.8 | 9.0±1.4 | 4.6±0.6 | 2.5±0.5 |
| | | + | | nt | 31.4±3.8 | 24.9±2.2 | 18.1±1.6 | 13.3±1.1 |
| | | | EC/TC= | 80 | 40 | 20 | 10 | 5 |
| 4 | $C_x$671 | − | | 28.0±1.8 | 29.6±4.2 | 21.7±1.3 | 13.3±2.6 | nt |
| | 2774 | − | | 53.3±1.3 | 48.9±0.9 | 39.3±8.8 | 23.1±1.2 | nt |
| 5 | $C_x$680 | − | | nt | 1.1±1.9 | 3.4±2.3 | 4.5±1.9 | 8.9±4.0 |
| | | + | | nt | 2.0±3.2 | −1.4±2.5 | 3.8±2.0 | 1.7±3.2 |
| | 2774 | − | | nt | 18.2±1.7 | 9.8±1.0 | 4.8±0.6 | 3.6±0.7 |
| | | + | | nt | 24.6±1.5 | 14.7±1.1 | 9.1±1.1 | 4.9±0.6 |

[a]Ovarian tumour cell line 2774 and various primary tissue lines ($C_x$) were used as target cells.
[b]Exogenous Hu IFN-α (160 u.ml$^{-1}$) added to the assay (+).
[c]Length of assay = 6 h.
[d]Effector cell/target cell ratio.
[e]Not tested.

**Figure 2** Competitive inhibition in NK assays against labelled (a) 2008 and (b) 2774 with unlabelled inhibitors: (△), 2008; (○), 2774; (●), K562; (■), SB. EC/TC = 50. Length of assay = 6 h.

**Table II** Cold target inhibition by K562 and ovarian carcinoma cells in NK assays against radiolabelled K562 and 2774 cells

| | | | % inhibition of NK[b] | | | | | | | |
|---|---|---|---|---|---|---|---|---|---|---|
| | | | K562 target cells[c] | | | | 2774 target cells[d] | | | |
| Exp. No. | IC[a] | IC/TC= | 8 | 4 | 2 | 1 | 8 | 4 | 2 | 1 |
| 1 | K562 | | 78 | 67 | 52 | 36 | 83 | 84 | 62 | 47 |
| | 2774 | | 46 | 20 | 14 | 11 | 73 | 56 | 52 | 39 |
| | $C_x$551 | | 17 | 13 | 4 | 3 | 65 | 45 | 39 | 28 |
| | $C_x$612 | | 9 | 10 | 1 | 2 | 13 | −2 | 0 | 6 |
| | SB | | 7 | 4 | −3 | −6 | nt[e] | nt | nt | nt |
| 2 | K562 | | 87 | 72 | 55 | 39 | 92 | 83 | 47 | 18 |
| | 2774 | | 27 | 31 | 21 | 10 | 88 | 90 | 71 | 17 |
| | $C_x$671 | | 10 | 9 | 6 | 7 | 36 | 21 | 14 | −5 |
| | $C_x$673 | | 10 | 9 | 7 | 2 | 6 | 1 | 4 | −5 |
| | SB | | 7 | 6 | 3 | nt | 17 | 2 | 4 | −6 |
| 3 | K562 | | 74 | 48 | 39 | 18 | 90 | 79 | 47 | 34 |
| | 2774 | | 36 | 18 | 21 | 2 | 82 | 82 | 62 | 51 |
| | $C_x$680 | | 50 | 42 | 36 | 24 | 89 | 86 | 78 | 67 |

[a]Unlabelled tumour cells added to NK assays as inhibitor cells (IC).
[b]% inhibition of radiolabelled target cell (TC) lysis by unlabelled inhibitor cells.
[c]Effector cell/target cell ratio = 10; length of assay, 3 h.
[d]Effector cell/target cell ratio = 40; length of assay, 6 h.
[e]Not tested.

**Table III** Morphology and NK activity of Percoll gradient fractionated effector cells

| Cell fraction (*fr*) | % cells recovered | % LGL[a] | % specific $^{51}Cr$ release (mean ± s.d.) K562 Target cells[b] | 2774 Target cells[c] |
|---|---|---|---|---|
| Input[d] | — | 4 | 15.8 ± 1.1 | 1.7 ± 0.5 |
| 1/2 | 4 | 15 | 12.7 ± 1.1 | nt[e] |
| 2/3 | 10 | 77 | 54.9 ± 2.1 | 10.6 ± 0.5 |
| 3/4 | 52 | 11 | 23.1 ± 1.2 | 0.9 ± 0.6 |
| 4/5 | 26 | 15 | 19.8 ± 1.4 | 1.1 ± 0.6 |
| 5 | 8 | 3 | 7.3 ± 1.6 | −1.4 ± 0.5 |

[a]Determined by Giesma staining.
[b]EC/TC = 5, 2-h assay.
[c]EC/TC = 20, 4-h assay.
[d]Input cells were mononuclear cells, depleted of monocytes by plastic adherence and nylon wool columns before fractionation. Peroxidase-positive cells < 1% in input population and all fractions.
[e]Not tested.

**Figure 3** Enhancement of NK against 2774 in Percoll fractionated effector cells by IFN-α. EC/TC = 5. Six-hour assay. (○), with IFN; (●), without IFN.

dependent cytolysis with increasing number of EC in NK assays. However, the degree of susceptibility varied among the TC, with most primary cell lines less sensitive to NK than long-term cell lines. Results from other studies have shown that fresh tumour cells are, in general, quite resistant to NK (Vose & Moore, 1980; Mantovani *et al.*, 1980). This is not necessarily a contraindication of the surveillance role proposed for NK, because resistance itself can be argued as a reason why tumours develop despite near normal NK activity in the peripheral blood of some cancer patients (Mantovani *et al.*, 1980; Kadish *et al.*, 1981). Resistance may be due either to lack of the appropriate NK recognition structure (or determinant) or properties inherent in the tumour cell membrane.

Competitive inhibition has been used to test for the presence of NK recognition determinants on TC. In most reports with long-term cultured cells, although heterogeneity exists, there is a good agreement between sensitive TC and effective inhibitors (Koren & Williams, 1978; Callewaert *et al.*). Roder and colleagues reported the isolation of a recognition structure from the murine YAC-1 cell line which has been shown to be expressed only by sensitive TC (Roder *et al.*, 1979). In our experiments with K562, 2008 and 2774, the cross inhibition among these TC indicates the presence of a common determinant. The inability of the insensitive SB target to compete with labelled, sensitive TC also agrees with this hypothesis (Callewaert *et al.*, 1979; Koren & Williams, 1978).

In experiments with primary tumour cell lines, two (Cx551) and Cx671) were significantly inhibitory to the cytolysis of 2774 but were ineffective in competition with K562 (Table II). Thus, there seems to be a quantitative difference in the common recognition determinant present on K562 and on 2774. As K562 is more sensitive to NK than 2774, which in turn shows greater sensitivity to NK than Cx551 and Cx671, it is possible that the number of NK recognition sites per cell also follows this order. As a result, at the inhibitor/target ratios we tested, although enough determinant sites were presented by the unlabelled Cx551 and by unlabelled Cx671 to compete with 2774, much less inhibition was observed in competition with labelled K562. No significant recognition sites could be detected on Cx612 or Cx673, as neither was inhibitory of NK against K562 or 2774 (Table II). This is consistent with our observation that Cx612 showed little susceptibility to NK. However, Cx680, which was totally insensitive to direct NK was a very effective inhibitor, comparable to the sensitive cell line 2774.

Similar results have been reported by Vose & Moore (1980). In their study, most fresh lung tumour cells were resistant to NK and were ineffective inhibitors in competitive inhibition assays using K562 as labelled TC. However, in 2/14 cell preparations they tested, significant inhibition against K562 was observed for resistant tumour cells. Inhibition of K562 cytolysis by fresh breast and lung tumour cells has also been reported by Ortaldo *et al.* (1977). Mantovani *et al.* (1980) also observed that NK-resistant, fresh ovarian tumour cells were inhibitory to NK against K562. Our results suggest that NK recognition determinant(s) may be expressed by some resistant tumour cells. Nonetheless, for the majority of the primary tumour cell lines tested, sensitive TC were generally better inhibitors. We have also shown that the presence of NK recognition determinant(s), although sometimes not detected by competitive inhibition assays using K562 as labelled TC, can be detected using the NK-sensitive 2774 cells as labelled TC.

Recent reports have clearly demonstrated that human NK as measured against K562 is mediated by LGL (Timonen *et al.*, 1981; Timonen & Saksela, 1981). As for the nature of EC in NK against other targets, contradictory results have been reported. De Vries *et al.* (1980) separated peripheral blood mononuclear cells according to their cell sizes by velocity sedimentation at unit gravity and tested NK activity in each fraction. The large cell fraction which contained mostly monocytes had little activity against any targets they tested. Although the fraction with small cells, mainly lymphocytes, was cytolytic for 2 leukaemic target cell lines, K562 and MOLT-4, very little activity against adherent target cell lines was observed. High activity against adherent cell lines was detected only in mixtures of both lymphocytes and monocytes. In contrast, de Landazuri *et al.* (1981) used Percoll gradients to fractionate PBL which had been depleted of monocytes by plastic adherence and nylon wool column, and found that the LGL-enriched fraction contained most of the NK activity against adherent as well as leukaemic targets. Addition of monocytes to the LGL-enriched EC did not cause any change in the NK activity. Neither did monocytes induce any NK activity in a high density lymphocyte fraction which consisted primarily of small lymphocytes. Furthermore, in the study by Uchida *et al.* (1982), peripheral blood monocytes obtained from patients after surgery were shown to suppress NK.

The results of our study are in accordance with those of de Landazuri *et al.* (1981). We found high NK activity only in EC populations with a high percentage of LGL, and the monocytes were not required for activity against either K562 targets or ovarian cell line 2774 and the other primary adherent line.

Enhancement of NK activity against 2774 by Hu IFN-$\alpha$ was observed primarily in fr2/3 of the Percoll gradients where the LGL content and original NK activity were highest. This is also consistent with recent reports that IFN-enhanced NK activity against both leukaemic targets and adherent targets was confined to LGL-enriched fractions (Timonen *et al.*, 1981; de Landazuri *et al.*, 1981). However, an interesting observation has been made by Vanky *et al.* (1980) that IFN only increases the NK activity against various allogeneic tumour cells but not against autologous tumour cells. Whether this is the case with ovarian tumour cells remains to be determined.

The results of our study suggest that natural cytotoxicity against adherent ovarian tumour cell lines is mediated by effector cells currently indistinguishable from those effective against standard NK targets though heterogeneity of effector cells in the LGL fraction can not be excluded. Further, the variable susceptibility of primary tumour cell lines to NK, is associated with the expression of NK recognition structure(s) on those cells and support the notion that NK cells may have a role in surveillance to tumour (Herberman *et al.*, 1979).

This work was supported by grants from the NCI, CA13070, CA14049 and CA23354. We are grateful to Mr. W. Travis for his help in preparing the Percoll gradients and for the reading of this manuscript.

### References

ARMSTRONG, J.A. (1971). Semi-micro, dye-binding assay for rabbit interferon. *Appl. Microbiol.*, **21**, 723.

BIRKE, C., PETER, H.H., LANGENBERG, U. & 7 others. (1981). Mycoplasma contamination in human tumor cell line: effect on interferon induction and susceptibility to natural killing. *J. Immunol.*, **127**, 94.

CALLEWAERT, D.M., KAPLAN, J., JOHNSON, D.F. & PETTERSON, Jr., W.D. (1979). Spontaneous cytotoxicity of cultured human cell lines mediated by normal peripheral blood lymphocytes. II. Specificity for target antigens. *Cell. Immunol.*, **42**, 103.

HERBERMAN, R.B., DJEU, J.Y., KAY, H.D. & 7 others. (1979). Natural killer cells: characteristics and regulation of activity. *Immunol. Rev.*, **44**, 43.

KADISH, A.S., DOYLE, A.T., STEINHAUER, E.S. & GHOSSEIN, N.A. (1981). Natural cytotoxicity and interferon production in human cancer: deficient natural killer activity and normal interferon production in patients with advance disease. *J. Immunol.*, **127**, 1817.

KAPLOW, L.S. (1965). Simplified myeloperoxidase stain using bezidine dihydrochloride. *Blood*, **26**, 215.

KOREN, H.S. & WILLIAMS, M.S. (1978). Natural killing and antibody-dependent cellular cytotoxicity are mediated by different mechanisms and by different cells. *J. Immunol.*, **121**, 1956.

DE LANDAZURI, M.O., LOPEZ-BOTET, M., TIMONEN, T., ORTALDO, J.R. & HERBERMAN, R.B. (1981). Human large granular lymphocytes: spontaneous and interferon-boosted NK activity against adherent and nonadherent tumor cell lines. *J. Immunol.*, **127**, 1380.

MANTOVANI, A., ALLAVENA, P., SESSA, C., BOLIS, G. & MANGIONI, C. (1980). Natural killer activity of lymphoid cells isolated from human ascitic ovarian tumors. *Int. J. Cancer*, **24**, 573.

ORTALDO, J.R., OLDHAM, R.K., CANNON, G.C. & HERBERMAN, R.B. (1977). Specificity of natural cytotoxic reactivity of normal human lymphocytes against a myeloid leukaemic cell line. *J. Natl Cancer Inst.*, **59**, 77.

PRETLOW, II, T.G., & PRETLOW, T.P. (1980). Separation of cells from tumors. *Contemp. Topics Immunobiol.*, **10**, 21.

RODER, J.C., ROSEN, A., FENYO, E.M. & TROY, F.A. (1979). Target-effector interaction in the natural killer cell system: isolation of target structure. *Proc. Natl Acad. Sci.*, **76**, 1405.

SHAU, H. & DAWSON, J.R. (1981). Enhancement of natural killing against ovarian tumor cells by humoral factor. *Fed. Proc.*, **40**, 1044.

SHAU, H. & DAWSON, J.R. (1982). Regulation of human natural killing by levamisole. *Cancer Immunol. Immunother.*, **13**, 24.

TIMONEN, T., ORTALDO, J.R. & HERBERMAN, R.B. (1981). Characteristics of human large granular lymphocytes and relationship to natural killer and K cells. *J. Exp. Med.*, **153**, 569.

TIMONEN, T. & SAKSELA, E. (1981). Isolation of human NK cells by density gradient centrifugation. *J. Immunol. Methods*, **36**, 285.

TIMONEN, T., SAKSELA, E., RANKI, A. & HAYRY, P. (1979). Fractionation, morphology and functional characterization of effector cells responsible for human natural killer activity against cell-line targets. *Cell. Immunol.*, **48**, 133.

UCHIDA, A., KOLB, R. & MICKSCHE, M. (1982). Generation of suppressor cells for natural killing activity in cancer patients after surgery. *J. Natl Cancer Inst.*, **68**, 735.

VANKY, F.T., ARGOV, S.A., EINHORN, S.A. & KLEIN, E. (1980). Role of alloantigens in natural killing: alogeneic but not autologous biopsy cells are sensitive for interferon-induced cytotoxicity of human blood lymphocytes. *J. Exp. Med.*, **151**, 1151.

VOSE, B.M. & MOORE, M. (1980). Natural cytotoxicity in man: susceptibility of freshly isolated tumor cells to lysis. *J. Natl Cancer Inst.*, **65**, 257.

DE VRIES, J.E., MENDELSOHN, J. & BONT, W.W. (1980). The role of target cells, monocytes, and Fc receptor-bearing lymphocytes in human spontaneous cell-mediated cytotoxicity and antibody-dependent cellular cytotoxicity. *J. Immunol.*, **125**, 396.

DE VRIES, J.E., MEYERING, M., VAN DONGREN, A. & RUMKE, P. (1975). The influence of different isolation procedures and the use of target cells from melanoma cell lines and cytotoxic effect of lymphocytes from healthy donors. *Int. J. Cancer*, **15**, 391.